# Clinical Efficacy and Imaging Observation of Three Surgical Approaches in Treatment of Thoracolumbar Fractures

**DOI:** 10.12669/pjms.39.3.3295

**Published:** 2023

**Authors:** Hui Li, Xiang-dong Liu, Wei-song Ma, Shun-yi Wang

**Affiliations:** 1 Hui Li, Department of Orthopedics, Baoding First Central Hospital, Baoding 071000, Heibei, P. R. China; 2 Xiang-dong Liu, Department of Orthopedics, Baoding First Central Hospital, Baoding 071000, Heibei, P. R. China; 3 Wei-song Ma, Department of Orthopedics, Baoding First Central Hospital, Baoding 071000, Heibei, P. R. China; 4 Shun-yi Wang, Department of Orthopedics, Baoding First Central Hospital, Baoding 071000, Heibei, P. R. China

**Keywords:** Thoracolumbar fracture, Paravertebral muscle space approach, Posterior median approach, Minimally invasive percutaneous approach, Clinical efficacy, Imaging

## Abstract

**Objective:**

To observe the efficacy and imaging of surgical treatment of thoracolumbar fractures via the paravertebral muscle space approach.

**Methods:**

A retrospective analysis was conducted on patients with thoracolumbar fractures receiving surgery in Baoding First Central Hospital from January 2019 to December 2020. According to different surgical approaches, they were divided into paravertebral approach group, posterior median approach group and minimally invasive percutaneous approach group. They received surgery via the paravertebral muscle space approach, posterior median approach and minimally invasive percutaneous approach, respectively.

**Results:**

Statistically significant differences were found in surgical duration, intraoperative bleeding volume, intraoperative fluoroscopy frequency, postoperative drainage volume and hospital stay among the three groups. One year after surgery, the VAS, ADL and JOA scores of the paravertebral approach group and the minimally invasive percutaneous approach group had statistically significant differences from the posterior median approach group (*P* < 0.05).

**Conclusion:**

For the surgical treatment of thoracolumbar fractures, the clinical efficacy of the paravertebral muscle space approach is superior to that of the traditional posterior median approach, and the clinical efficacy of the minimally invasive percutaneous approach is similar to that of the posterior median approach. All the three approaches can effectively improve the postoperative function and pain symptoms of patients without increasing the incidence of complications. Compared with the posterior median approach, the surgery via the paravertebral muscle space and minimally invasive percutaneous approaches presents shorter surgical duration, less bleeding and shorter hospital stay, which is more conducive to postoperative recovery of patients.

## INTRODUCTION

Thoracolumbar fractures are a common type of spinal fractures in clinic, accounting for about 50% of spinal fractures,^1^ which are mostly caused by violence, and mainly occur in young adults and the elderly. With the development of transportation and construction industries, the incidence of thoracolumbar fractures has increased significantly. Pedicle-screw internal fixation is the main method for thoracolumbar fractures, during which the posterior median approach is a classic surgical approach for the treatment of thoracolumbar fractures. However, extensive dissection and long-term traction of the paravertebral muscles are required during the surgery, which is prone to ischemia, necrosis and denervation of the paravertebral muscles, resulting in postoperative flatback deformity, intractable low back pain and stiffness.^2^-^4^ To reduce the occurrence of these complications, a variety of approaches have emerged. Among them, the paravertebral muscle space and minimally invasive percutaneous approaches are gradually emerging surgical approaches for thoracolumbar fractures in recent years.

The paravertebral muscle space approach, also known as the Wiltse approach, adopts the muscle space between the multifidus muscle and the longissimus muscle as the surgical approach to insert the pedicle screw under direct vision. This approach can well expose the articular process and transverse process of T10-S1 vertebrae. The minimally invasive percutaneous approach is a surgical approach to insert a working cannula between soft tissues, and place pedicle screws through the working channel. Both of the above two surgical approaches can avoid extensive dissection, destruction and injury of the paravertebral muscles during operation, which is conducive to rapid postoperative recovery of patients.^5^-^7^ To investigate the clinical efficacy and safety of the above three surgical approaches, this study analyzed the efficacy of open reduction and internal fixation via the posterior median approach, paravertebral muscle space approach and minimally invasive percutaneous approach in the treatment of single-segment thoracolumbar fractures, and explored the clinical efficacy and changes in imaging indexes of the paravertebral muscle space approach in the treatment of single-segment thoracolumbar fractures.

## METHODS

A total of 105 patients with thoracolumbar fractures in Baoding First Central Hospital were selected from January 2019 to December 2020. The study was approved by the Institutional Ethics Committee of Baoding First Central Hospital at July 17, 2020, and it conforms to the Declaration of Helsinki formulated by the World Medical Association (WMA).

### Inclusion criteria:

- Single-segment thoracolumbar fractures (T11-L1) confirmed by imaging, and fresh fractures confirmed by MRI;
- Excluded pathological fractures, such as tumor-associated fractures;
- No surgical contraindications;
- No nerve injury or decompression;
- Follow-up time > 1 year.

### Exclusion criteria:

- Patients with surgical contraindications or severe underlying diseases, and intolerance to surgery;
- Patients refusing surgical treatment;
- Patients with old thoracolumbar fractures;
- Patients with pathological fractures;
- Patients accompanied by severe osteoporosis;

Follow-up time < 1 year. According to different surgical approaches, the patients were divided into paravertebral approach group (receiving surgery via the paravertebral muscle space approach), posterior median approach group (receiving surgery via the open posterior median approach) and minimally invasive percutaneous approach group (receiving surgery via the minimally invasive percutaneous approach), with 35 patients in each group. No statistically significant differences were found in the general data among the three groups before surgery (*P* > 0.05), suggesting comparability, as seen in Table-I.

**Table-I:** Comparison of general data among the three groups (n = 35).

Group	Gender (Male/Female)	Age (Year)	Cause of injury (traffic accident injury/ falling injury/others)	Site of injury (thoracic vertebra/ lumbar vertebra)
Paravertebral approach group	22/13	57.03±11.15	14/16/5	17/18
Posterior median approach group	25/10	57.69±7.54	15/14/6	21/14
Minimally invasive percutaneous approach group	21/14	58.46±8.68	15/15/5	19/16
F/c^2^	1.850	0.209	0.304	0.291
p	0.581	0.811	0.990	0.631

In the paravertebral approach group, the surgery was carried out via the paravertebral muscle space approach. Under general anesthesia, the patients were in the prone position, and routine disinfection and towel laying were performed. The diseased vertebral segments were determined and marked under C-arm fluoroscopy. A posterior median incision was made centered on the fractured vertebra, to cut open and expose the lumbodorsal fascia layer by layer. The lumbodorsal fascia (or the superficial pectoralis and dorsalis muscle) was cut open longitudinally, and the space between the longissimus muscle and the multifidus muscle was bluntly separated, to expose the articular process and the root of the transverse process. Pedicle screws were inserted into the injured vertebra and adjacent vertebral pedicles. After confirming the good position of pedicle screws by fluoroscopy, a pre-bent connecting rod was placed to stretch and reduce the injured vertebra. With the good reduction of the injured vertebra, the good position of pedicle screws, and tightened screws confirmed under fluoroscopy, a drainage tube was placed after washing the wound, followed by suturing layer by layer, sterile dressing and fixation. Finally, the surgery was completed.

The posterior median approach group was treated with internal fixation via the open posterior median approach. Under general anesthesia, the patients were in the prone position, followed by fluoroscopic marking, disinfection and towel laying. Then, a posterior median incision with length of about 15-20 cm was made, the skin, subcutaneous layer and lumbodorsal fascia were cut open successively, and the bilateral paravertebral muscles were dissected along both sides of the spinous process. After exposing the articular process and transverse process, two pedicle screws were inserted in the injured vertebra and its upper and lower vertebrae, respectively, with a total of six pedicle screws inserted. The other treatment was the same as the paravertebral approach group.

In the minimally invasive percutaneous approach group, the minimally invasive percutaneous approach was adopted, and the patients were in the prone position under general anesthesia, followed by routine disinfection and towel laying. Under fluoroscopy, the position of the injured vertebra was determined, the surface projections of the pedicles of the injured vertebra and its adjacent upper and lower vertebrae was located, and the percutaneous puncture site was confirmed. Afterwards, a positioning needle was placed percutaneously, and a 1.5-2.5 cm incision was made with the positioning needle as the center, to cut open the skin and subcutaneous tissues successively. Subsequently, a working cannula was inserted along the positioning needle for gradual expansion. With the help of the working channel, hollow pedicle screws of appropriate size were inserted step by step. After confirming the good position of pedicle screws under fluoroscopy, a pre-bent connecting rod was placed and a distractor was used for distraction and reduction. Then, with the good reduction of the injured vertebra, the good position of pedicle screws, and tightened screws confirmed under fluoroscopy, a drainage tube was placed after washing the wound, followed by suturing layer by layer, sterile dressing and fixation. Finally, the surgery was completed.

### Observation indexes:

Perioperative indexes included surgical duration, intraoperative blood loss, intraoperative fluoroscopy frequency, postoperative drainage volume and hospital stay. Imaging indexes included kyphosis Cobb angle and the relative height of the anterior edge of the injured vertebrae before and one year after surgery. The kyphosis Cobb angle was measured with the angle between the tangents of the upper and lower vertebral endplates of the injured vertebra. The relative height of the anterior edge of the injured vertebrae = (actual height of the vertebral anterior edge/reference height of the vertebral anterior edge) × 100%. The VAS, ADL and JOA scores before surgery, six months and one year after surgery were statistically compared among the three groups. The clinical efficacy one year after surgery was assessed using the modified MacNab criteria. The complications of the three groups during follow-up were recorded and compared, such as incisional infection, internal fixation breakage and looseness, and cerebrospinal fluid leakage.

### Statistical methods:

Statistical analysis was carried out using SPSS 22.0. Normality analysis was conducted firstly for inter-group measurement data. The measurement data conforming to the normal distribution were all expressed as mean ± standard deviation (*χ̅*±*S*), and compared between groups using one-way ANOVA. The enumeration data were expressed as *n* (%), and compared between groups by the c² test. *P* < 0.05 was considered as statistically significant.

## RESULTS

Perioperative indexes Statistically significant differences were found in surgical duration, intraoperative bleeding volume, intraoperative fluoroscopy frequency, postoperative drainage volume and hospital stay among the three groups (all *P* < 0.05). The surgical duration was the shortest in the paravertebral approach group, while the longest in the posterior median approach group. The intraoperative fluoroscopy frequency was the highest in the minimally invasive percutaneous approach group, while the lowest in the paravertebral approach group and the posterior median approach group. The intraoperative bleeding volume and postoperative drainage volume were the smallest in the minimally invasive percutaneous approach group, while the largest in the posterior median approach group. The hospital stay was the shortest in the paravertebral approach group and the minimally invasive percutaneous approach group, while the longest in the posterior median approach group, as shown in Table-II.

**Table-II:** Comparison of perioperative indexes among the three groups (*χ̅*±*S*).

Group	Surgical duration (min)	Intraoperative fluoroscopy frequency (time)	Intraoperative bleeding volume (ml)	Postoperative drainage volume (ml)	Hospital stay (d)
Paravertebral approach group	91.29±7.51^a^	4.17±1.01	215.57±9.30^a^	89.71±17.53^a^	6.83±0.98^a^
Posterior median approach group	134.00±7.84	4.09±0.89	290.43±9.19	145.00±23.10	8.20±1.91
Minimally invasive percutaneous approach group	111.33±5.86^a,b^	6.17±1.27^a,b^	136.43±9.74^a,b^	78.00±10.30^a,b^	6.71±0.83^a^
F	318.255	42.617	2343.146	141.932	13.578
p	0.000	0.000	0.000	0.000	0.000

***Notes:***

a compared with the posterior median approach group, p<0.05; ^b^ compared with the paravertebral approach group, p<0.05.

Imaging indexes Before treatment, the kyphosis Cobb angle and the relative height of the anterior edge of the injured vertebrae had no statistically significant differences among the three groups (all *P* > 0.05). After treatment, the kyphosis Cobb angle and the relative height of the anterior edge of the injured vertebrae in the three groups were significantly improved compared with those before surgery (all *P* < 0.05). The improvement in the relative height of the anterior edge of the injured vertebrae after surgery was the most significant in the posterior median approach group, and showed statistically significant differences among the three groups (*P* < 0.05). However, the Cobb angle presented no statistically significant differences among the groups (*P* > 0.05) (Table-III).

**Table-III:** Comparison of Cobb angle and relative height of the anterior edge of the injured vertebrae before and after surgery (*χ̅*±*S*)

Group	Cobb angle before surgery (°)	Cobb angle after surgery (°)	Relative height of the anterior edge of the injured vertebrae before surgery (%)	Relative height of the anterior edge of the injured vertebrae after surgery (%)
Paravertebral approach group	22.20±0.35	6.77±2.26	48.48±7.36	89.08±6.53^a^
Posterior median approach group	22.38±0.40	7.43±1.87	49.55±7.38	92.71±6.28
Minimally invasive percutaneous approach group	22.28±0.45	7.51±2.23	47.69±6.78	85.32±6.42^a,b^
F	1.692	1.276	0.588	11.630
p	0.189	0.284	0.557	0.000

***Notes:***

a compared with the posterior median approach group, p<0.05; ^b^ compared with the paravertebral approach group, p<0.05.

Improvement in pain and neurological function Before surgery, no statistically significant differences were detected in VAS, ADL or JOA score among the three groups (all *P* > 0.05). With the increase in follow-up time, the VAS scores of the three groups were significantly lower than those before surgery, while the ADL and JOA scores increased gradually, and had statistically significant differences at different time points in the groups (all *P* < 0.05). One year after surgery, the VAS, ADL and JOA scores of the paravertebral approach group and the minimally invasive percutaneous approach group had statistically significant differences from the posterior median approach group (all *P* < 0.05), as presented in Table-IV.

**Table-IV:** Comparison of VAS, ADL and JOA scores among the three groups at different time points before and after surgery (*χ̅*±*S*).

Group	Time point	VAS score	ADL score	JOA score
Paravertebral approach group	Before surgery	7.51±0.51	45.09±2.58	10.57±3.40
	6 months after surgery	3.51±0.51	71.86±3.77	19.49±2.73
	1 year after surgery	1.23±0.70*	85.71±3.73*	24.40±1.97*
Posterior median approach group	Before surgery	7.31±0.47	44.57±3.18	11.14±2.91
	6 months after surgery	3.54±0.56	72.46±4.20	19.66±2.29
	1 year after surgery	1.69±0.63	83.00±3.43	22.51±2.05
Minimally invasive percutaneous approach group	Before surgery	7.46±0.51	45.03±3.52	10.23±3.09
	6 months after surgery	3.40±0.50	70.80±3.91	18.51±2.34
	1 year after surgery	1.06±0.68*	85.66±2.98*	23.54±1.75*

***Notes:***

* compared with the posterior median approach group during the same time period, p < 0.05.

*Clinical efficacy and complications* The excellent and good rates of efficacy assessed by the modified MacNab criteria were 82.86%, 80.00% and 74.28% in the three groups, respectively, without statistically significant differences (*P* > 0.05) (Table-V). After surgery, the incisions of the three groups were all healed at the one stage, without infection. No severe complications occurred during the 1-year follow-up.

**Table-V:** Comparison of MacNab-based excellent and good rates among the three groups 1 year after surgery [*n*, (%)].

Group	*n*	Excellent	Good	Intermediate	Poor	Excellent and good rate
Paravertebral approach group	35	21(60.00)	8(22.86)	4(11.43)	2(5.71)	29(82.86)
Posterior median approach group	35	19(54.29)	9(25.71)	5(14.29)	2(5.71)	28(80.00)
Minimally invasive percutaneous approach group	35	16(45.71)	10(28.57)	6(17.14)	3(8.57)	26(74.28)
c^2^						0.805
p						0.669

## DISCUSSION

The thoracolumbar vertebrae are located at the thoracolumbar junction, and the spine transits from the relatively fixed thoracic vertebrae to the relatively active lumbar vertebrae. When the axial load of the thoracolumbar segments increases due to traffic accidents, falls from height, etc., the thoracolumbar vertebrae are prone to fractures.^8^ Most thoracolumbar fractures need active surgical treatment to reduce the disability rate of patients. However, there are still controversies about the optimal surgical opportunity and method for thoracolumbar fractures, and there is still no guidance for the selection of surgical approach.^9^ A study has shown that in the cases of obvious endplate involvement, posterior ligament complex injuries or severe vertebral fractures in the fractured vertebrae evidenced by imaging, surgery is needed to prevent progressive kyphosis and intractable low back pain.^10^ In addition, early surgery for patients with thoracolumbar fractures can shorten their hospital stay and bed-rest time, which can reduce the occurrence of relevant complications. Moreover, surgery can restore the normal physiological curvature of the spine, which is conducive to the early rehabilitation of patients.^11^-^15^

Surgical treatments for thoracolumbar fractures are various, mainly including surgery via the anterior approach and posterior approach. The posterior median approach is the most common and classic approach, which is characterized by clear anatomy, simple operation, and ability to be used for laminectomy and intervertebral fusion. Nevertheless, extensive dissection and long-term traction of the paravertebral muscles are required during the surgery via the posterior median approach, which will lead to ischemia, necrosis and denervation of the paravertebral muscles. After surgery, patients will present intractable low back pain and flatback deformity.^16^-^18^ Additionally, the angle and accuracy of pedicle screw insertion will be affected by the shielding of the paravertebral muscles.^19^,^20^ To reduce the iatrogenic injury of the paraspinal muscles and increase the accuracy of pedicle screw insertion, clinicians have tried a variety of surgical approaches, among which the paravertebral muscle space approach and minimally invasive percutaneous approach are the most successful. Via the paravertebral muscle space approach, the articular process is entered through the muscle space between the longissimus muscle and the multifidus muscle, and the pedicle screw is inserted under direct vision, which avoids the dissection and traction of the paravertebral muscles, reduces intraoperative bleeding loss and shortens surgical duration.^21^-^23^ Moreover, it also preserves the integrity of posterior spinal muscle-ligament complexes, and less damage the biomechanical stability of the spine.^24^,^25^ Because of the small incision and the assistance of the working channel, minimally invasive percutaneous internal fixation has less damage to the soft tissues, reduces the stimulation and interference to the nerves and blood vessels, and avoids denervated muscle atrophy.

In our study, the difference in the postoperative relative height of the anterior edge of the vertebra between the paravertebral approach group and the posterior median approach group was not statistically significant, but both were superior to the minimally invasive percutaneous approach group (*P* < 0.05), indicating that the minimally invasive percutaneous approach has a slightly poor effect on the recovery of the vertebral height. The paravertebral approach group and the minimally invasive percutaneous approach group were superior to the posterior median approach group in surgical duration, intraoperative bleeding volume, postoperative drainage volume and hospital stay (*P* < 0.05), but the intraoperative fluoroscopy frequency in the minimally invasive percutaneous approach group was higher than that in the paravertebral approach group and the posterior median approach group (*P* < 0.05). The VAS, ADL and JOA scores of the paravertebral approach group and the minimally invasive percutaneous approach group were better than those in the posterior median approach group one year after surgery (*P* < 0.05), suggesting that the two minimally invasive surgical approaches reduce the occurrence of intractable low back pain, paravertebral muscle stiffness and other complications due to the damage to muscles and soft tissues during the surgery. One year after surgery, no difference was found in clinical efficacy between the paravertebral approach group and the minimally invasive percutaneous approach group (*P* > 0.05), that is, there were no differences in ADL, JOA or VAS scores. Compared with the minimally invasive percutaneous approach, the paravertebral muscle space approach increases intraoperative bleeding volume, but reduces intraoperative fluoroscopy frequency and shortens surgical duration.

### Limitations of the study:

It includes small sample size and short follow-up time, which affect the level of evidence to a certain extent. We look forward to a long-term follow-up study with a large sample size in the future.

### Authors’ Contributions:

**HL and** **SYW** designed this study and prepared this manuscript, and are responsible and accountable for the accuracy or integrity of the work.

**XDL** collected and analyzed clinical data.

**WSM** significantly revised this manuscript.
